# Renal function-adapted D-dimer cutoffs in combination with a clinical prediction rule to exclude pulmonary embolism in patients presenting to the emergency department

**DOI:** 10.1007/s11739-023-03521-3

**Published:** 2024-02-14

**Authors:** Simon Flueckiger, Svenja Ravioli, Carlos Buitrago-Tellez, Michael Haidinger, Gregor Lindner

**Affiliations:** 1 Department of Internal and Emergency Medicine, Buergerspital Solothurn, Solothurn, Switzerland; 2 Department of Emergency Medicine, King’s College Hospital NHS Foundation Trust, London, UK; 3 Department of Radiology, Buergerspital Solothurn, Solothurn, Switzerland; 4 Department of Emergency Medicine, Inselspital, University Hospital Bern and University of Bern, Bern, Switzerland; 5 Klinik für Allgemeine Innere und Notfallmedizin, Bürgerspital Solothurn, Schöngrünstrasse 42, 4500 Solothurn, Switzerland

**Keywords:** Emergency, D-dimer, Pulmonary embolism, Renal insufficiency

## Abstract

D-dimer levels significantly increase with declining renal function and hence, renal function-adjusted D-dimer cutoffs to rule out pulmonary embolism were suggested. Aim of this study was to "post hoc" validate previously defined renal function-adjusted D-dimer levels to safely rule out pulmonary embolism in patients presenting to the emergency department. In this retrospective, observational analysis, all patients with low to intermediate pre-test probability receiving D-dimer measurement and computed tomography angiography (CTA) to rule out pulmonary embolism between January 2017 and December 2020 were included. Previously defined renal function-adjusted D-dimer cutoffs (1306 µg/l for moderate and 1663 µg/l for severe renal function impairment) were applied to determine sensitivity, specificity, negative and positive predictive values. One thousand, three hundred sixty-nine patients were included of which 229 (17%) were diagnosed with pulmonary embolism. The estimated glomerular filtration rate (eGFR) was ≥ 60 ml/min in 1079 (79%), 30–59 ml/min in 266 (19%) and < 30 ml/min in 24 (2%) patients. Only three patients (1.1%) with an eGFR < 60 ml/min had a D-dimer level < 500 µg/l. There was a significant correlation between D-dimer and eGFR (*R* = − 0.159, *p* < 0.001). Calculated on the standard D-dimer cutoff value of 500 µg/l, sensitivity of D-dimer testing was 97% for patients with an eGFR ≥ 60 ml/min and 100% for those with 30–60 ml/min, while specificity decreased in patients with renal function impairment. A negative predictive value of 0.99 as a premise to safely rule out pulmonary embolism was achieved by applying a D-dimer cutoff of 1480 µg/l for eGFR 30–59 ml/min and 1351 µg/l for eGFR < 30 ml/min. The findings of this study underline that application of renal function-adapted D-dimer levels in combination with a clinical prediction rule appears feasible to rule out pulmonary embolism. Out of the current dataset, renal function-adjusted D-dimer cutoffs to rule out pulmonary embolism were slightly different compared to previously defined cutoffs. Further studies on a larger scale are needed to validate possible renal function-adjusted D-dimer cutoffs.

## Introduction

Venous thromboembolism (VTE), as represented by deep vein thrombosis and pulmonary embolism, constitutes the third most common cardiovascular disease with an annual incidence of approximately 100–200 per 100,000 persons [1, 2]. Acute pulmonary embolism is considered the most serious clinical presentation of VTE with a variety in clinical presentation ranging from incidental findings to sudden death [2–4]. The seriousness of pulmonary embolism is depicted by a study in which more than 300,000 deaths were attributed to the condition in six countries of the European Union [2].

Current guidelines by the European Society of Cardiology (ESC) on the diagnosis and treatment of acute pulmonary embolism suggest testing for age-adapted D-dimer levels to rule out presence of pulmonary embolism in low to intermediate risk patients as stratified by calculation of a clinical prediction rule such as the Wells score or the revised Geneva score [5–10].

The D-dimer-based approach in addition to the before-mentioned clinical prediction rules is reasonable as further diagnostic testing such as computed tomography angiography (CTA) or ventilation–perfusion scans are not necessary in case of negative D-dimer results, which in turn saves time-consuming and potentially harmful diagnostic tests.

D-dimer is a highly specific marker for the degradation of cross-linked fibrin. On the other hand, fibrin itself is elevated in a broad spectrum of conditions such as cancer, infections or necrosis resulting in a low positive predictive value of D-dimer for the diagnosis of pulmonary embolism [5]. Studies have indicated that the specificity of D-dimer decreases steadily with age to about 10% in patients aged 80 years or older [11]. For that reason, age-adjusted D-dimer cutoff values were proposed and tested safely in various studies [12].

In a small study in patients with suspected pulmonary embolism, the specificity of D-dimer for diagnosis of pulmonary embolism was significantly reduced in patients with renal insufficiency [13]. This finding could be interpreted as a consequence of an increased fibrin breakdown in renal insufficiency as was suggested in the past [14]. In a recently published, larger study, D-dimer levels significantly correlated with renal retention parameters and inversely with estimated glomerular filtration rate (eGFR) [15]. A similar pattern has been found for patients with diabetic nephropathy [16]. Additionally, specificity for D-dimer in the diagnosis of pulmonary embolism declined significantly while sensitivity remained high or even increased with worsening renal function [15]. Almost no patients with an eGFR below 60 ml/min had D-dimer levels below 500 µg/l, which is considered the overall reference range [15, 17]. A different research group confirmed these findings in a subsequent study [18]. These findings suggest that eGFR-adapted D-dimer cutoffs could result in less need for further diagnostic procedures to rule out pulmonary embolism. This approach is of particular interest considering the high and potentially increasing prevalence of chronic kidney disease and especially of Kidney Disease Outcome Quality Initiative (KDOQI) Stage III (eGFR 30–59 ml/min) [19, 20]. Therefore, the application of renal function-adjusted D-dimer cutoffs might reduce not only costs due to unnecessary diagnostics, but even more importantly reduce radiation exposure and the risk for developing contrast-induced nephropathy particularly in patients with eGFR < 45 ml/min [21, 22].

Due to the comparably low number of patients with renal insufficiency, especially those with higher grade renal insufficiency (KDOQI Stage IV and V) in our primary study, calculation of cutoffs resulting in a post-test probability for presence of pulmonary embolism < 1% was not possible [15, 23]. Performance of a larger follow-up study allowed for calculation of renal function-adjusted D-dimer cutoffs resulting in a negative predictive value of > 99% [17]. However, the study was single-centered. Hence, the aim of the present study was to evaluate the applicability of the previously defined renal function-adjusted D-dimer cutoffs to rule out pulmonary embolism in conjunction with a clinical prediction rule in a different setting.

## Methods

All patients presenting to the Department of Emergency Medicine of the Buergerspital Solothurn, Switzerland, between January 2017 and December 2020 who received measurements of D-dimer and serum creatinine as well as a CTA scan to rule out pulmonary embolism were included. Patients were screened on basis of the database of our radiology department. We excluded all patients with age under 18 years, patients with written verbal or oral withdrawal of consent and those under treatment with an anticoagulant (phenprocoumon, rivaroxaban, edoxaban, apixaban, heparin or low-molecular weight heparin) in therapeutic intent at the time of presenting to the Emergency Department. None of the patients included was under treatment with either dabigatran or warfarin.

### Data acquisition

Of all patients included in the study, the following parameters were obtained from electronic patient charts: sex, age, presence and location of pulmonary embolism as detected by CTA and documented in the corresponding report. If not documented in the admission chart, the Wells score was calculated based on the medical records available at time of admission. Data on creatinine, D-dimer and C-reactive protein (CRP) from serum analysis were gathered. Glomerular filtration rate was estimated according to the Chronic Kidney Disease Epidemiology Collaboration Formula (CKD-EPI) [24]:

$$\begin{aligned} {\text{eGFR}} = & 141 \times \min \left( {{\text{Scr}}/\kappa ,1} \right)^{\alpha } \times \max \left( {{\text{Scr}}/\kappa ,1} \right)^{ - 1.209} \times 0.993^{{{\text{Age}}}} \\ & \times 1.018 \, \left[ {{\text{if}}\;{\text{female}}} \right] \times 1.159\left[ {{\text{if}}\;{\text{Black}}} \right], \\ \end{aligned}$$

where Scr is serum creatinine (mg/dl), *к* is 0.7 for women and 0.9 for men, is − 0.329 for women and − 0.411 for men, min and max representing the minimum and maximum of Scr/к or 1, respectively).

D-dimer was measured in our central laboratory by use of the HemosIL D-dimer HS Instrumentation Laboratory (Milano, IT, reference range < 243 µg/l, sensitivity > 99%, specificity 38.4%, negative predictive value > 99%), an automated latex enhanced immunoassay. The system had been updated in October 2017 to HemosIL D-dimer HS 500 with a consecutive change in the reference range to 500 µg/l (sensitivity > 99%, specificity 45.1%, negative predictive value > 99%).

### Statistical analysis

Data analysis was performed using R (Version 0.98.707 for Mac OS X) [25]. Continuous data are presented as median with interquartile range (IQR) between first and third quartile and categorical variables as absolute counts and percentage. Distribution of continuous variables was assessed using Q–Q normal plots. For intergroup comparison of categorical variables, Fisher's exact test was performed with the Freeman–Halton extension for more than two groups. Continuous variables were compared using Mann–Whitney U and Kruskal–Wallis tests with Bonferroni correction as appropriate. Correlation between two categorical variables was measured by Kendall's т coefficient and a multivariate regression analysis was performed for several risk factors on D-dimer levels with logarithmic transformation applied when reasonable. Sensitivity, specificity, negative (NPV) and positive (PPV) predictive values as well as likelihood ratios were calculated for D-dimer using standard equations. A NPV of ≥ 99% was considered save and a two-sided confidence level of 95% (*p*-value ≤ 0.05) statistically significant. Net reclassification improvement (NRI) was calculated according to standard formula described in Pencina et al. [26].

### Cost-effectiveness analysis

In terms of cost-effectiveness, the monetary costs of performed CTA-scans that could have safely (with a post-test probability of < 1%) been omitted were calculated. We therefore applied the renal function-adjusted D-dimer cutoffs as derived from the study population on patients with low to moderate pre-test probability (i.e., Wells score < 6.5) but D-dimer levels above the age-adjusted cutoff. For cost evaluation, tax points as listed in the Swiss TARMED catalogue for the technical procedure as such and for the assessment by an approved radiologist were cumulated.

### Ethical considerations

The study was approved by the local Ethics Committee (Ethikkommission Nordwest- und Zentralschweiz, www.eknz.ch, project-ID 2021-02255) in Switzerland. Due to its retrospective design and the large number of patients included in the study, the need for obtaining informed consent was waived.

## Results

Within the investigated period, 1376 Patients received CTA to rule out pulmonary embolism. Seven were excluded due to therapeutic anticoagulation, which left 1369 patients included in the final analysis. Median age was 66 years (IQR 54–79) and 732 patients were female (53%). Median serum creatinine level was 78 µmol/l (62–94), corresponding to a medium eGFR of 83 ml/min (64–102) computed by the CKD-EPI formula as presented in the “Methods” Section 24. patients (2%) had a CKD-EPI eGFR < 30 ml/min and 266 (19%) an eGFR 30–59 ml/min. Of 1079 patients (79%) with an eGFR ≥ 60 ml/min, 559 patients had normal renal function (eGFR > 90 ml/min). Of the included 1369 patients, 229 were diagnosed with pulmonary embolism by CTA (17%) with localization described as peripheral or subsegmental in 42 (19%), segmental in 76 (33%), paracentral in 37 (16%) and central in 74 (32%) cases. There was no significant difference between the three eGFR subclasses (eGFR ≥ 60 ml/min, eGFR 30–59 ml/min, eGFR ≤ 30 ml/min) regarding frequency of pulmonary embolism (*p*-value 0.3). D-dimer levels below 500µg/l were found in three (1.1%) patients with moderate (eGFR of 30–59 ml/min) and in none with severe (eGFR < 30 ml/min) kidney function impairment. Furthermore, no patients with documented pulmonary embolism on CTA scan and eGFR < 60 ml/min had a D-dimer level < 500 µg/l. Table 1 gives an overview on distribution of patients with pulmonary embolism and median of D-dimer stratified for CKD-EPI eGFR. Details on test performance of D-dimer to rule out pulmonary embolism are given in Table 2. The corresponding test accuracy was 26% for patients with an eGFR ≥ 60 ml/min and 21% for patients with an eGFR of 30–59 ml/min, the area under the receiver operating curve was 0.78 (confidence interval CI 0.75–0.81) for all patients.

**Table 1:** Findings on D-dimer levels and prevalence of pulmonary embolism separated according to renal function

CKD-EPI eGFR	≥ 60 ml/min	30–59 ml/min	< 30 ml/min
Number of patients	1079	266	24
D-Dimer			
Median (IQR)	1096 (465–1727)	1925 (376–3473)	1686 (792–2579)
< 500µg/l	115 (10.7%)	3 (1.1%)	0
Pulmonary embolism	173 (16%)	53 (20%)	3 (12.5%)

*CKD-EPI eGFR* Chronic Kidney Disease Epidemiology Collaboration estimated glomerular filtration rate; *IQR* interquartile range

**Table 2:** Test-performance of D-dimer to rule out pulmonary embolism in all patients and in those with eGFR ≥ 60 ml/min and 30–59 ml/min, respectively

	All patients	eGFR ≥ 60 ml/min	eGFR 30–59 ml/min
Sensitivity	0.98 (CI 0.96–1.0)	0.97 (CI 0.95–1.0)	1.0 (CI 1.0–1.0)
Specificity	0.1 (CI 0.08–0.12)	0.12 (CI 0.1–0.14)	0.014 (CI 0.0–0.03)
PPV	0.18 (CI 0.16–0.2)	0.17 (CI 0.15–0.2)	0.2 (CI 0.15–0.25)
NPV	0.96 (CI 0.92–0.99)	0.96 (CI 0.91–0.99)	1.0 (CI 1.0–1.0)
PLR	1.09 (CI 1.06–1.12)	1.1 (CI 1.07–1.14)	1.01 (CI 1.0–1.03)
NLR	0.22 (CI 0.09–0.54)	0.24 (CI 0.1–0.58)	0
Accuracy	0.25	0.26	0.21

*CKD-EPI eGFR* Chronic Kidney Disease Epidemiology Collaboration estimated glomerular filtration rate; *CI* 95% confidence interval; *PPV* positive predictive value; *NPV* negative predictive value; *PLR* positive likelihood ratio; *NLR* = negative likelihood ratio

Details on population characteristics regarding Wells score for pulmonary embolism are presented in Tables 3 and 4. The prevalence of pulmonary embolism for Wells risk classes low (2%), moderate (36%) and high (54%) were similar to previous findings in existing literature [7] (Table 3). Applying the YEARS criteria for pulmonary embolism [27], three (1.3%) cases of pulmonary embolism could have been excluded without conducting a CTA. Table 4 shows odds ratios for the constituent criteria of the Wells score. There was no statistical significance for hemoptysis (*p*-value 0.57) and active malignancy (*p*-value 0.69), whereas the other criteria each met statistical significance (*p*-value < 0.01).

**Table 3:** Wells score for patients with suspected pulmonary embolism (PE)

Wells risk class for PE	Total patients	Percent (%)	Patients with PE	Prevalence
Low risk (Wells score < 2.0)	818	59.8	18	0.02
Moderate risk (Wells score 2.0–6.0)	492	35.9	179	0.36
High risk (Wells score > 6.0)	59	4.3	32	0.54

**Table 4:** Odds ratio of the different Wells score criteria for patients with suspected pulmonary embolism (PE)

Wells criteria	Odds ratio for PE	*p*-value
Clinical sign of DVT	3.16	< 0.001
Alternative diagnosis less likely than PE	32.27	< 0.001
Heart rate > 100/min	1.5	0.005
Recent surgery or immobilization	2.22	< 0.001
Previous PE or DVT	3.92	< 0.001
Hemoptysis	1.34	0.57
Malignancy	1.1	0.69

*DVT* deep vein thrombosis

**Fig. 1:**
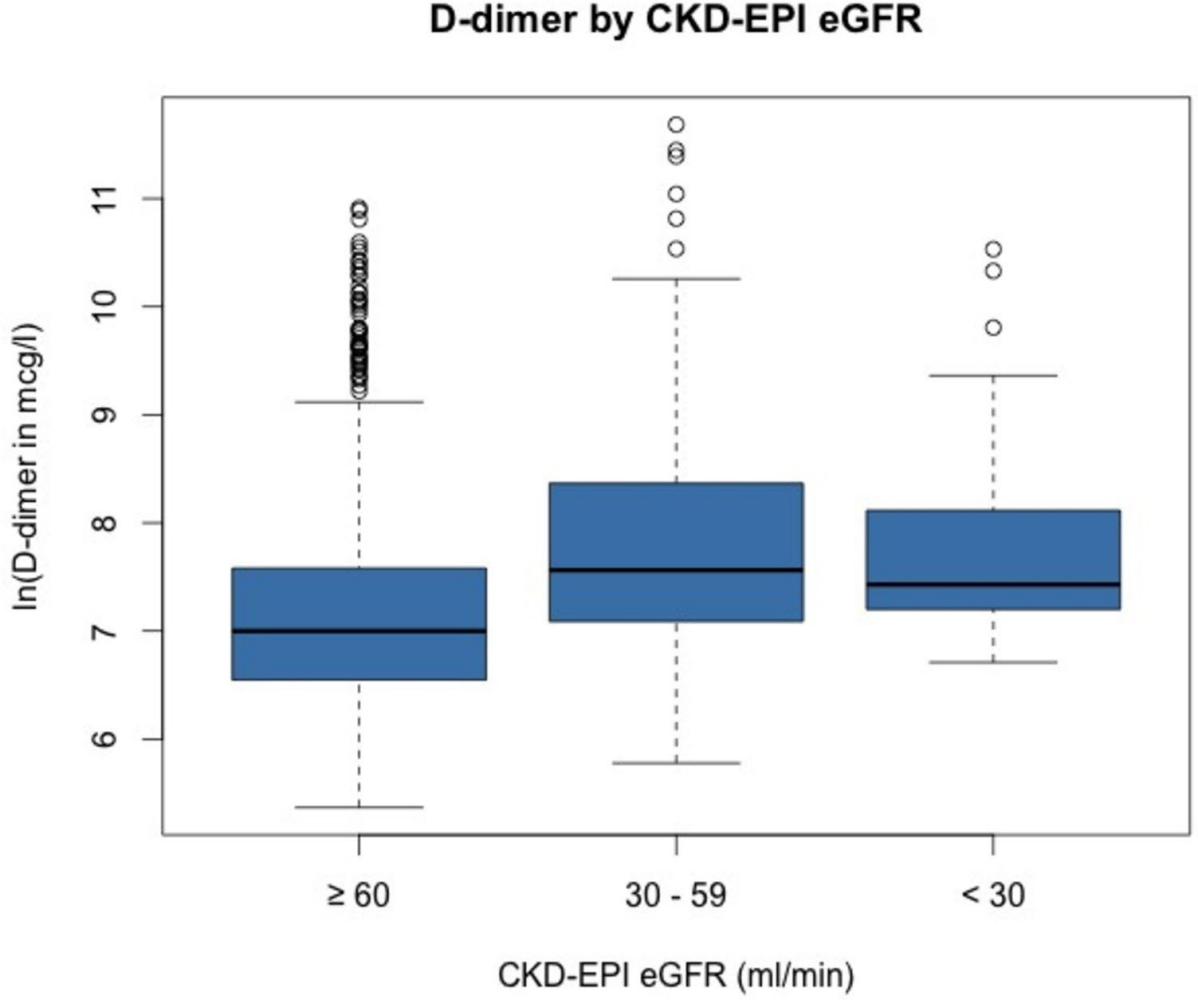
Boxplot for D-dimer distribution in patients with Chronic Kidney Disease Epidemiology Collaboration estimated glomerular filtration rate (CKD-EPI eGFR) ≥ 60 ml/min, 30–60 ml/min and < 30 ml/min, respectively. The upper whisker represents the top 25% D-dimer values and the lower whisker the bottom 25%. For overview purposes, D-dimer values are given as natural logarithm

**Fig. 2:**
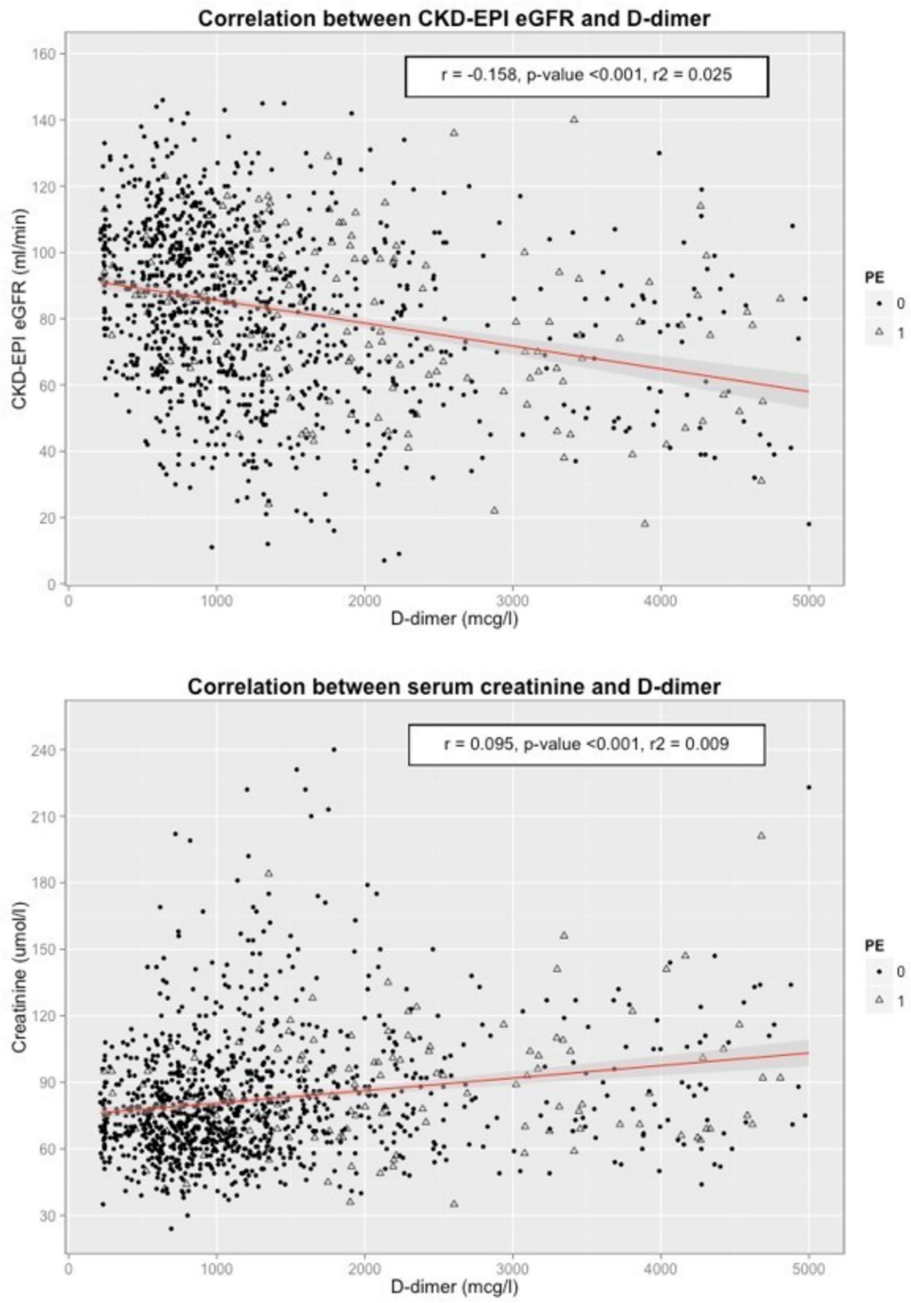
Correlation between D-dimer and Chronic Kidney Disease Epidemiology Collaboration estimated glomerular filtration rate (CKD-EPI eGFR, upper figure) or serum creatinine (bottom figure). Triangles represent cases with and dots cases without documented pulmonary embolism (PE) in a computer tomographic angiography. For a better overview, only cases with D-dimer < 5000 µg/l and serum creatinine < 300 µmol/l are depicted, whereas the given correlation coefficients were calculated on the entire study population

Table 5 presents the results of a multivariate regression analysis on logarithmic D-dimer. Age, documented pulmonary embolism on CTA scan, logarithmic CRP and eGFR, active malignancy and pregnancy or perinatal postpartum states (defined as the first week after birth) all showed significant correlation with D-dimer levels, particularly significant in the four first mentioned (*p*-value < 0.001). In the univariate linear regression analysis, the laboratory parameters serum creatinine (*R* = 0.096), eGFR (*R* = − 0.159) and CRP (*R* = 0.104) correlated significantly with D-dimer levels (*p*-value < 0.001). The correlation for D-dimer with serum creatinine and eGFR is depicted in Fig. 1. Furthermore, D-dimer levels were significantly lower in patients with eGFR ≥ 60 ml/min compared to patients with eGFR 30–59 ml/min and eGFR < 30 ml/min (*p*-value < 0.001), respectively. However, no significant difference in D-dimer levels was found comparing patients with eGFR 30–59 ml/min to those with eGFR < 30 ml/min (*p*-value 0.87) and the median of D-dimer was even higher in patients with eGFR 30–59 ml/min (1925 µg/l vs. 1686 µg/l) with a larger variance as shown in Table 1 and Fig. 2.

**Table 5:** Results of a multivariate regression analysis on D-dimer for several population characteristics and risk factors

ln(D-Dimer)	Regression coefficient	Standard error	*p*-value
Age	0.012	0.002	< 0.0001
Female sex	− 0.07	0.048	0.128
Pulmonary embolism	0.959	0.065	< 0.0001
Malignancy	0.237	0.085	0.0056
Pregnancy / perinatal	0.918	0.362	0.0114
ln(eGFR)	− 0.3467	0.0788	< 0.0001
ln(CRP)	0.0297	0.0087	0.0009

*ln* natural logarithm; *CRP* C-reactive protein; *eGFR* Chronic Kidney Disease Epidemiology Collaboration (CKD-EPI) estimated glomerular filtration rate; *Perinatal period* first 7 days after giving birth

We identified cutoff values of D-dimer required to rule out pulmonary embolism for CKD-EPI eGFR as well as for CRP by achieving a negative predictive value of 0.99 and 0.95, respectively. The results are presented in Table 6. The D-dimer cutoff values adjusted for CRP showed a continuous, significant increase with increasing CRP levels. Although the D-dimer cutoffs for eGFR < 60 ml/min were significantly higher than 500 µg/l, decreasing D-dimer from patients with eGFR 30–59 ml/min to those with eGFR < 30 ml/min were found. The D-dimer cutoff values differed accordingly with 1480 µg/l for eGFR 30–59 ml/min and 1351 µg/l for eGFR < 30 ml/min to achieve a NPV of at least 0.99. If D-dimer cutoffs calculated by Pfortmueller and coworkers [17] were applied on the present study population, the sensitivity was 0.98 and the NPV 0.988 for moderately (D-dimer cutoff 1306 µg/l) and 0.67 and 0.917, respectively, for severely (D-dimer cutoff 1663 µg/l) impaired kidney function. Applying the D-dimer cutoffs derived in our study to rule out pulmonary embolism on a post-test probability level of < 1% (1480 µg/l and 1351 µg/l, respectively), 109 negative CTA scans would have been conducted on patients with an eGFR of 30–59 ml/min (instead of 187) and 14 on patients with an eGFR of < 30 ml/min (instead of 20). Thus, the costs (488.- CHF per CTA scan) that could have been avoided by applying the presently derived D-dimer cutoffs instead of the established age-adapted ones amounted to approximately 38,000.- CHF for patients with eGFR 30–59 ml/min (78) and 3000.- CHF for those with eGFR < 30 ml/min (6). While all patients with pulmonary embolism and eGFR < 60 ml/min had received a CTA by applying the established, age-adapted D-dimer cutoff, one case would have been missed by applying the cutoffs derived in this study (which in any case are higher than the age-adapted ones, so an additional age-adaption was not required). We only included cases with a Wells score < 6.5 points in this analysis, as ESC guidelines suggest to conduct a CTA scan without measuring D-dimer levels for a high clinical probability (Wells score > 6 points) [10]. A net reclassification improvement analysis for D-dimer only, applied to all patients with an eGFR < 60 ml/min, yielded a NRI of 0.154 for the standard, age-adapted cutoffs and a NRI of 0.87 for the renal function-adapted cutoffs derived in this study. A direct comparison between the standard and the renal function-adapted cutoffs showed a NRI of 0.358 in favor of the renal function-adapted cutoffs.

**Table 6:** Cutoff values for D-dimer adjusted to Chronic Kidney Disease Epidemiology Collaboration (CKD-EPI) estimated glomerular filtration rate (eGFR) and C-reactive protein (CRP)

	Cutoff for D-dimer (µg/l)	Sensitivity	Specificity	PPV	NPV
*eGFR*					
30–59 ml/min	< 1480	0.98	0.46	0.31	0.99
	< 1683	0.89	0.54	0.32	0.95
	< 1306*	0.98	0.39	0.29	0.988
< 30 ml/min	< 1351	1.0	0.33	0.18	0.99 (1.0)
	< 1638	0.67	0.48	0.15	0.9 (0.91)
	< 1663*	0.67	0.52	0.17	0.917
*CRP*					
≥ 50 mg/l	< 242	1.0	0.01	0.2	0.99 (1.0)
	< 633	0.97	0.13	0.21	0.95
≥ 100 mg/l	< 824	1.0	0.19	0.18	0.99 (1.0)
	< 1829	0.81	0.63	0.28	0.95
≥ 150 mg/l	< 1224	1.0	0.38	0.14	0.99 (1.0)
	< 3299	0.6	0.81	0.23	0.95

*PPV* positive predictive value; *NPV* negative predictive value

*Marked = D-dimer cutoffs as calculated in Pfortmueller CA, Linder G et al. [23] applied on the present study population

## Discussion

In the present study, the correlation of D-dimer levels and renal function showed a similar pattern compared to previous studies conducted on this topic and the results regarding applicability of renal function-adapted D-dimer cutoffs appear promising. One concerning issue, however, is the lack of further increase of D-dimer levels from patients with moderate (CKD-EPI eGFR 30–59 ml/min) to patients with severe (eGFR < 30 ml/min) kidney function impairment which stands in contrast to the findings of previous studies. Thus, the eGFR-adjusted D-dimer cutoffs derived from this study for achieving a post-test probability of < 1% differ from the ones of previous studies [15, 17] in such, as the cutoff value for patients with an eGFR < 30 ml/min was below the one for patients with an eGFR 30–59 ml/min and lower than the ones previously calculated. In view of these results, it needs to be considered that the very low number of patients with pulmonary embolism and severely impaired kidney function (3) and the correspondingly large variance pose a significant limitation to statistical analysis for this patient collective. Still, this result did not come by surprise as CTA scans are avoided whenever possible in patients with eGFR < 30 ml/min to reduce exposure to potentially nephrotoxic contrast. Therefore, the results of the present study implicate the need for further clinical trials with significantly larger study populations yet with a similar prevalence of pulmonary embolism. The calculated, possibly saved costs (41,000.- CHF) by omitting CTA scans on basis of renal function-adjusted D-dimer cutoff and the increased NRI further support investigation of this approach which has also been stated in a previous cost-effectiveness analysis [28]. Since proof-of-concept was promising for application of renal function-adjusted D-dimer levels in the distinctively larger collective of patients with eGFR of 30–60 ml/min, it could be reasonable to focus on this patient group exclusively in further studies. For those patients with severe renal impairment, the newest guidelines suggest lung scintigraphy (planar ventilation/perfusion V/Q scan or V/Q SPECT) as an alternative imaging technique to diagnose pulmonary embolism [10]. However, limited availability of infrastructure and application knowledge as well as the comparatively high costs have prevented a broader use of V/Q scans so far and the percentage of V/Q scans performed to diagnose pulmonary embolism even declined over the last 10 years in various regions [29]. Therefore, efforts of health care systems are needed to improve availability of lung scintigraphy for specific patient subgroups.

Wells score was calculated for each included patient to assess clinical pre-test probability of pulmonary embolism. Wells score was chosen over the revised Geneva score due to its slightly higher test efficiency and lower prediction failure [30]. The prevalence of pulmonary embolism stratified by the risk class of the Wells score was similar to values documented before. However, no statistical significance regarding correlation of pulmonary embolism and presence of active malignancy or reported hemoptysis was found in the present study. This might at least partly be explained by the small number of cases presenting with those two features. Furthermore, Wells score does not differentiate with regard to type of malignancy, even though the relative risk for pulmonary embolism does significantly vary among different types of cancers [31].

As the mutual dependency of acute phase response and hemostasis is well known [32], a univariate linear regression between D-dimer and CRP was performed, finding a significant increase in D-dimer levels with increasing CRP. CRP-adjusted D-dimer cutoff values were derived for CRP ≥ 50 mg/l, ≥ 100 mg/l and ≥ 150 mg/l in order to examine a possible increase in specificity of D-dimer as prognostic tool. The obtained results suggest a cutoff adaptation similar to the one for patient age, which is already widely applied [11]. This underlines the need for further research on that matter on a large-scale basis to reevaluate possible CRP-adjusted D-dimer cutoffs.

The main limitations of the present study are its non-prospective design and the low number of patients with eGFR < 30 ml/min. Because of the latter, the validity of our results regarding patients with severely impaired kidney function is quite limited. Additionally, the assay for D-dimer measurement changed during the investigated period with a consecutive shift of reference range and specificity and hence, a distortion of D-dimer levels between the period before and after the adjustment cannot be ruled out. Furthermore, Wells scores had been calculated retrospectively on basis of clinical presentations as documented in electronic patient charts with potential errors due to biased assumptions regarding certain symptoms and incomplete medical history. And since the decision to measure D-dimer levels and to conduct a CT-scan relied on the experience of the responsible clinician, there is a theoretical risk for a sampling error in such, as patients who did not receive these diagnostics would not have been screened by our selection criteria. Thus, the patients who in fact had an elevated Wells score but, nevertheless, did not receive a CT-scan were not included into our study.

To conclude, our study confirmed the previously described inverse correlation of D-dimer values with eGFR, at least up to a certain degree of renal function impairment. Hence, the specificity of D-dimer levels to rule out pulmonary embolism decreases significantly with declining renal function. We therefore encourage further investigation in a larger study population with the long-term objective to define renal function-adjusted D-dimer cutoffs to rule out pulmonary embolism.

## Data Availability

All data was obtained from a electronic patient chart and are therefore still available.

## Abbreviations

CI: Confidence interval
CKD-EPI: Chronic Kidney Disease Epidemiology Collaboration
CRP: C-reactive protein
CTA: Computed tomography angiography
DVT: Deep vein thrombosis
eGFR: Estimated glomerular filtration rate
ESC: European Society of Cardiology
IQR: Interquartile range
KDOQI: Kidney Disease Outcome Quality Initiative
ln(): Natural logarithm
NLR: Negative likelihood ratio
NPV: Negative predictive value
NRI: Net reclassification improvement
PE: Pulmonary embolism
PLR: Positive likelihood ratio
PPV: Positive predictive value
VTE: Venous thromboembolism
V/Q scan: Ventilation/perfusion scan
